# Pathogen-induced dormancy in liquid limits gastrointestinal colonization of *Caenorhabditis elegans*

**DOI:** 10.1080/21505594.2023.2204004

**Published:** 2023-04-25

**Authors:** Liyang Zhang, Vyshnavi Gade, Natalia V. Kirienko

**Affiliations:** Department of BioSciences, Rice University, Houston, TX, USA

**Keywords:** *C. elegans*, *P. aeruginosa*, liquid-based infection model, pathogen-induced dormancy

## Abstract

Colonization is generally considered a prerequisite for infection, but this event is context-dependent, as evidenced by the differing ability of the human pathogen *Pseudomonas aeruginosa* to efficiently colonize *Caenorhabditis elegans* on agar but not in liquid . In this study, we examined the impact of the environment, pathogen, host, and their interactions on host colonization. We found that the transition to a liquid environment reduces food uptake by about two-fold. Also expression of specific adhesins was significantly altered in liquid-based assays for *P.* *aeruginosa*, suggesting that it may be one factor driving diminished colonization. Unexpectedly, host immune pathways did not appear to play a significant role in decreased colonization in liquid. Although knocking down key immune pathways (e.g. *daf-16* or *zip-2*), either alone or in combination, significantly reduced survival, the changes in colonization were very small. In spite of the limited bacterial accumulation in the liquid setting, pathogenic colonization was still required for the virulence of *Enterococcus faecalis*. In addition, we found that a pathogen-induced dormancy was displayed by *C.* *elegans* in liquid medium after pathogen exposure, resulting in cessation of pharyngeal pumping and a decrease in bacterial intake. We conclude that poor colonization in liquid is likely due to a combination of environmental factors and host-pathogen interactions. These results provide new insights into mechanisms for colonization in different models, enabling pathogenesis models to be fine-tuned to more accurately represent the conditions seen in human infections so that new tools for curbing bacterial and fungal infections can be developed.

## Introduction

The rapid spread of antibiotic resistance and the stagnation of novel antibiotic development have created the looming threat of an untreatable bacterial pestilence [1–3]. The opportunistic Gram-negative bacterium *Pseudomonas aeruginosa* is a noteworthy example of the development of this problem. Notorious for its intrinsic and acquired antimicrobial resistance, *P. aeruginosa* strains that shrug off all available treatments are being identified with increasing frequency. Although this threat currently remains somewhat manageable, *P. aeruginosa* is responsible for thousands of deaths and millions in healthcare costs each year [4–7]. It is increasingly clear that new treatments are needed. There is a growing consensus that these new therapies should focus on disarming, rather than simply killing, the pathogen, as this may help to stave off the rapid development of resistance.

Developing these treatments requires a more nuanced understanding of the pathogenic mechanisms used by bacterial agents, allowing therapy to act more like a scalpel than a cannon. To address this need, scientists have created a variety of infection models for *P. aeruginosa* in a range of model organisms, including the nematode *Caenorhabditis elegans*. *C. elegans* has several advantages for studying host-pathogen interactions, including a relatively complex complement of differentiated organ systems, substantial conservation of innate immune pathways with humans, well-developed tool sets for genetics and cell biology, and adaptability of the host to various environments [8–13]. Using *C. elegans* as a host, several different *P. aeruginosa* pathogenesis models were developed, such as Slow Killing, Fast Killing, Lethal Paralysis, and Red Death, each highlighting different aspects of *P. aeruginosa* virulence [14]. One limitation of most of these models is that worms are reared on agar plates but most high-throughput chemical screening approaches depend on liquid-based techniques to identify compounds of pharmacological interest. One liquid-based pathogenesis model, called Liquid Killing, was derived from the Slow Killing assay to address this problem [15]. For similar reasons, liquid-based *C. elegans* infection assays were developed for other pathogens, including the Gram-positive bacteria *Enterococcus faecalis* and *Enterococcus faecium*, and the pathogenic yeast *Candida albicans* [16–18]. These assays have been used for screening libraries of small molecules, genetic mutants to identify host and pathogen factors that regulate these interactions, and panels of pathogenic isolates collected from patients [18–22]. In the process of characterizing these assays, it has become clear that there are differences between these liquid-based assays and their agar-based predecessors. For example, *P. aeruginosa* Liquid Killing showed limited intestinal colonization, which differed markedly from the Slow Killing assay where colonization is a key determinant of virulence [15]. The mechanism(s) leading to differing levels of colonization have remained elusive.

*C. elegans* has evolved multiple layers of defense to ensure its survival in a natural environment, where it lives in rotting fruit, eating a variety of microbes present in the decaying matter [23]. Organismal level avoidance responses allow worms to evade infectious bacteria [24]; physical defenses, including the body’s exterior cuticle, pharyngeal grinder, and intestinal epithelium, serve as barriers to infection; and innate immune pathways enable worms to mount an immune response to infections, promoting survival [25]. Besides the host defense adopted by most animals under unfavorable conditions, larval C. *elegans* are able to enter a specialized, metabolically dormant stage called the “dauer” [26]. Dauer larvae are physiologically specialized to be very stress resistant and consume virtually no food, and worms can maintain this state for long periods (up to 120 days, or six times their regular lifespan). Adult worms can also enter a dormant, stress-induced protective sleep-like stage or quiescence [27]. Entry into this state is triggered by the epidermal growth factor (EGF) receptor in the ALA neuron, which cooperates with downstream neuropeptides in response to several stimuli, including noxious heat, cold, hypertonicity, and tissue damage [28]. This dormant stage is relatively short (several minutes to a few hours at a time) compared to the dauer stage, but is characterized by substantially less pharyngeal pumping and suppressed locomotion [27]. Little is known about whether developed adult worms undergo a persistent dormant state when encountering stress, especially pathogens.

This study aims to characterize the difference between agar- and liquid-based *C. elegans* pathogenesis models and to identify the mechanism(s) used by worm to resist pathogenic colonization in a liquid setting. We found that colonization by *P. aeruginosa*, *E. faecalis*, or *C. albicans* was compromised in liquid settings. This was caused by a complex combination of decreased feeding, activation of host defense due to the liquid milieu, downregulation of bacterial adhesins, and pathogen-induced host dormancy. Duration of exposure to the pathogen played a role in colonization for some microbes (e.g. *E. faecalis*), but this effect was smaller. Initial bacterial cell density and nutritional composition of the liquid condition did not appear to affect colonization. Unexpectedly, despite these hurdles, the pathogenesis of *E. faecalis* was partially dependent on colonization in the liquid-based model. The detailed mechanism is currently unknown and requires further study. This report shows that while some features of liquid-based infection models (e.g. decreased colonization, increased host dormancy, *etc*.) may be common to multiple pathogens, others may be model-specific. Understanding context-depending pathogenicity is an important step for designing successful screens for novel small molecule treatments and the identification of relevant bacterial targets for the development of future therapeutics.

## Materials and methods

### Strains

Bacterial strains: Unless otherwise noted, *C. elegans* were reared on *E. coli* OP50. For RNAi studies, worms were reared on *Escherichia coli* HT115(DE3) carrying RNAi-expressing plasmids [29]. For pathogenesis assays, *P. aeruginosa* PA14, PA14:dsRed, *Enterococcus faecalis* OG1RF, *E. faecalis* OG1RF:GFP, *Candida albicans* fRS26, and *C. albicans* fRS26:GFP were used.

*C. elegans* strains: The temperature-sensitive sterile strain *glp-4(bn2)* was used for all experiments [30], unless otherwise noted. Immunocompromised ERT061 [*zip-2(tm4248)*] worms were used to assess the effect of the immune pathways knockdown on colonization [31]. To synchronize worms, eggs were isolated from gravid adults by hypochlorite worm bleach and then hatched in S Basal at room temperature. Synchronized L1 larvae were maintained on nematode growth media (NGM) plates seeded with *E. coli* OP50 or RNAi bacteria *E. coli* HT115 at 25 °C until they reached late L4 stage. For RNAi experiments, *E. coli* was inoculated into 5 mL LB medium with 100 μg/mL carbenicillin (Thermo Fisher Scientific) and incubated at 37°C overnight with 225 rpm shaking. The next day, bacteria were evenly seeded onto LB plates containing 1 mM IPTG and 100 μg/mL carbenicillin. Plates were incubated at room temperature for 24 hours before use. 2,000 synchronized L1 larvae were transferred onto 6 cm RNAi plates and grown at room temperature overnight and at 25°C for 48 hours prior to use for experiments.

### Slow killing and liquid killing assays

Slow Killing (SK) and Liquid Killing (LK) assays were performed as previously reported [32]. For SK assays, briefly, 70 μL of *P. aeruginosa* PA14 overnight culture was spread evenly onto a 3.5 cm SK plate (3 g NaCl, 3.5 g peptone, 18 g agar in 972 mL ddH_2_O, after autoclave, add 1 mL 1 M MgSO_4_, 25 mL 1 M KH_2_PO_4_, pH 6, 1 mL 1 M CaCl_2_, and 1 mL 5 mg/mL cholesterol in ethanol) and grown at 37 °C for 24 hours, followed by 25 °C for another 24 hours. ~50 young adult *C. elegans* were placed on each plate and incubated at 25 °C. Worms were scored daily; dead worms were removed from assay plates. Each of the three biological replicates contained three technical replicates. A log-rank test was used to determine the statistical significance of the Slow Killing assay.

For LK assays, Liquid Killing medium (30% SK media in S Basal with 1 mM MgSO_4_, 1 mM CaCl_2_, and 5 μg/mL cholesterol) was mixed with *P. aeruginosa* PA14 (final OD_600_: 0.03) and then 50 µL were added to each well of a 384-well plate. For *E. faecalis* or *C. albicans*, 10% BHI in S Basal with 5 μg/mL cholesterol was mixed with pathogens (final OD_600_: 0.02) and 50 µL were added to each well of a 384-well plate. 20 *glp-4(bn2)* worms were added into each well using a COPAS FlowPilot sorter (UnionBiometrica). At least 20 wells were used for each condition. The plate was covered with an air-permeable membrane and incubated at 25 °C until a predetermined time. Bacteria were washed from the plate with S Basal 5 times. Worms were stained with 50 μL of 0.7 μM Sytox® Orange solution (Life Technologies) per well for 16–24 hours. Excess stain was washed away three times with S Basal, and then fluorescence and bright-field micrographs were captured with a Cytation5 multimode reader (BioTek). Three biological replicates were performed.

For dormancy measurements, Liquid Killing assays were performed as above except that bright-field micrographs of worms were taken every 2 hours for 24 or 48 hours. Worms that appeared unbent and lacking a sinusoidal appearance were deemed dormant. Three biological replicates were performed.

For experiments using PQS, it was added into the LK medium at a final concentration of 100 μM. Control samples used DMSO only. Micrographs of aggregated cells and infected worms were captured using a fluorescent microscope (Zeiss Axio Imager M2). Three biological replicates were performed.

### Fluorescence-based colonization quantification

Young adult *glp-4(bn2)* worms were infected in modified SK assays using~70 worms/plate and *P. aeruginosa* PA14:dsRed, *E. faecalis* OG1RF:GFP, or *C. albicans* fRS26:GFP in place of the unmarked pathogen or in modified LK assays where pathogens expressing fluorescent markers replaced their non-fluorescent counterparts. At set intervals, infected worms were collected into the 1.5 mL Eppendorf tubes and washed with 1 mL S Basal 3 times. After the final wash, 100 μL S Basal with 1% levamisole (Acros Organics) was added to paralyze *C. elegans*. Pictures were captured under a fluorescent microscope (Zeiss Axio Imager M2) using appropriate filter sets. Fluorescence intensity for each worm was measured manually. At least 50 worms were imaged for each condition in each biological replicate. Three biological replicates were performed.

### Worm colony-forming unit (CFU) assay

Microbial colony-forming unit assays were performed as previously reported [33]. In short, ~70 adult *glp-4(bn2)* worms were infected on plates containing each pathogen of interest at 25°C for predetermined times. 15 worms from each plate were collected into 1.5 mL Eppendorf tubes containing 100 μL of 1% levamisole and washed with 1 mL of S Basal 6 times to remove residual bacteria. 10 µL of S Basal from the final wash were plated onto an LB plate as a blank control. The volume was aspirated to 100 μL and then 100 μL of S Basal and 300 μL of zirconium beads (Fisher Scientific, 1.0 mm) were added, and tubes were vortexed vigorously for 1 minute at maximum speed to break open worms. Lysates were serially diluted 5-fold and dilutions were plated onto 10 cm LB plates. The colonies were counted after a 24-hour incubation at 37 °C and colony-forming units (CFUs) per each worm were calculated. Each of three biological replicates included three technical replicates.

### Food uptake measurement

For solid media assays, 1 mL Slow Killing agar media supplemented with 3 μg/mL riboflavin (vitamin B2) was added into each well of a 24-well plate. Each well was seeded with 10 μL heat-killed OP50, and 5 μL fluorescent beads (Fluoresbrite® Polychromatic Red Microspheres 1.0 µm. *Polysciences, Inc*.). 20 worms were picked onto each well and were incubated at 25 °C.

For liquid media assays, 35 μL Slow Killing media supplemented with 3 μg/mL riboflavin, 10 μL heated OP50, 5 μL fluorescent beads, and 20 worms in S Basal were added to each well. The plate was covered with an air-permeable membrane and incubated at 25 °C. After 24-hour incubation, worms were collected and washed with S Basal 3 times. *C. elegans* were paralyzed in 1% levamisole (Acros Organics) in S Basal. Fluorescent and bright-field micrographs were collected using a fluorescence microscope (Zeiss Axio Imager M2) using appropriate filter sets. Fluorescence intensity for each worm was manually calculated. Each of the three biological replicates included three technical replicates.

### Quantitative PCR

The 100 μL of *P. aeruginosa* pellet was collected from the SK plate and the LK medium for RNA extraction, respectively. The RNA was extracted using Trizol with BCP as a phase-separating agent, and cDNA was synthesized using a cDNA synthesis kit (New England BioLabs). qPCR was conducted in a CFX-96 real-time thermocycler (Bio-Rad) using SYBR green AzuraQuant Fast Green Fastmix (Azura). Primer sequences can be found in Table 1. Fold changes were calculated using a ΔCt method with 30S ribosomal protein S12 as a housekeeping gene. Each of the three biological replicates contained three technical replicates.

**Table 1.** Quantitative PCR primers used in this project.

Gene	Primer	Sequence	Source
*pilI*	Forward	ATCGTCGATGAGGTGTTCGG	this study
	Reverse	CGCCATGAATGAAGGGTTGC	this study
*pilJ*	Forward	AACGCAGGCAATCTTTTCGC	this study
	Reverse	GTCATGGTTCGACTGGGTGT	this study
*pelA*	Forward	TGGAACAGCCAGGTAATGGAC	this study
	Reverse	AAGCTGTCCAGGGTATCGAG	this study
*pelB*	Forward	TCTGCCGCTGACAAGCATC	this study
	Reverse	CGCTGGGCATGAATACCTCT	this study
*pa1L*	Forward	GATCTGCCACGATGCGTTTT	this study
	Reverse	ACCCGGTATTGACCGGAATG	this study
*lecB*	Forward	GGAGTGTTCACCCTTCCCG	this study
	Reverse	CGACGGTTTCGTTGTTGACC	this study
*rpsL*	Forward	CTGCGTAAGGTATGCCGTGT	this study
	Reverse	GTTGTGACCTTCACCACCGA	this study

## Liquid-Killing with the supernatant of *E. faecalis*

Freshly-streaked *E. faecalis* was inoculated into BHI medium at an initial OD_600_ of 0.1 and incubated at 25 °C for 3 or 6 days. Spent growth medium was centrifuged at 10,000 g and then supernatant was filtered through a 0.22 μm syringe-tip filter. 50 μL of the resulting supernatant was then added to each well of a 384-well plate and then 20 young adult *glp-4(bn2)* worms were sorted into each well. Half of the wells had *E. faecalis* added at a final OD_600_ of 0.02. After 5 days of incubation at 25 °C, survival was evaluated using Sytox® Orange. Bright-field and fluorescent micrographs were collected using a Cytation5 multi-mode plate reader (BioTek). Three biological replicates were performed.

## Results

### Pathogenic colonization is compromised in liquid-based pathogenesis models

**Figure 1.**
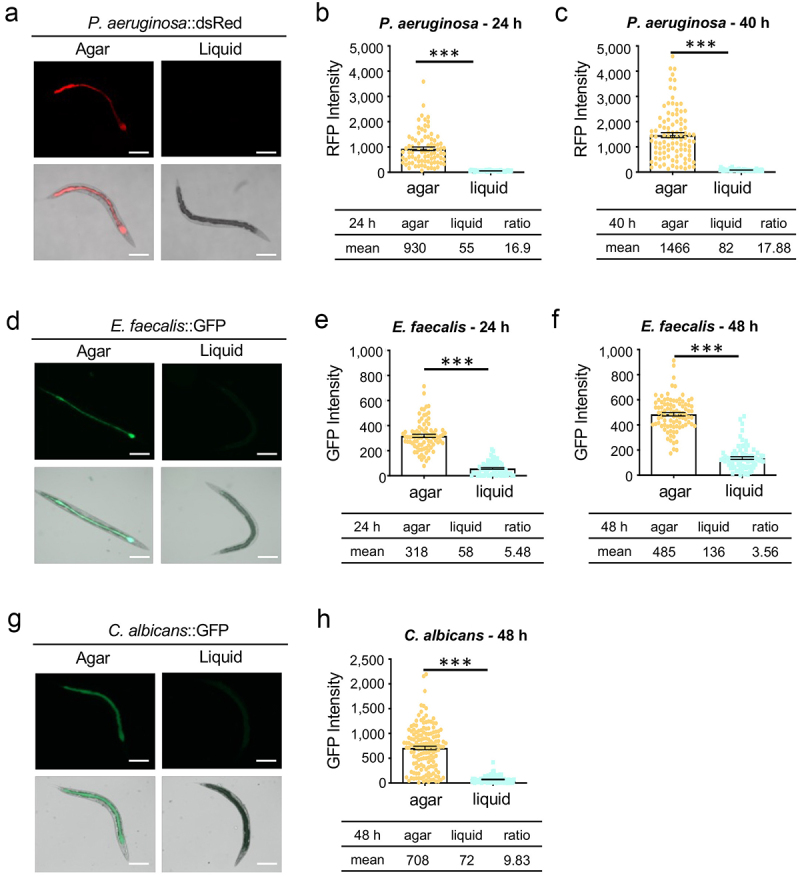
Pathogen colonization is diminished in a liquid context. Young adult worms were fed *P.* *aeruginosa* PA14:dsRed (a), *E.* *faecalis* OG1RF:GFP (d), or *C.* *albicans* fRS26:GFP (g) for 24 hours on agar plates or in liquid media. Micrographs show the accumulation of fluorescent pathogens in the intestinal lumen. Scale bar 200 μm. (b, c, e, f, h) Fluorescence intensity from the pathogens within worms was quantified. Three biological replicates, of at least 50 worms each, were performed. Error bars represent SEM. *p* values were determined using Student’s *t*-test. ***p < 0.001.

We previously observed significantly greater intestinal colonization of *C. elegans* when it feeds on a bacterial lawn of *P. aeruginosa* (Slow Killing) than when worms are exposed to *P. aeruginosa* in a liquid medium (Liquid Killing) [15]. To confirm that this effect was not an artifact of the shorter exposure time commonly used for liquid pathogenesis assays but instead reflects changes in host-pathogen interaction, colonization of worms exposed to *P. aeruginosa* in either Liquid Killing or in Slow Killing assays was measured at both early (24 hours) and late (40 hours) periods during the Liquid Killing assay. At both times, colonization was significantly lower (~17-fold lower at 24 hours and ~18-fold lower at 40 hours) in Liquid Killing (Figure 1a–c). Since colonization was already effectively established by 24 hours in worms feeding on *P. aeruginosa* on agar and worms typically die between 44 hours and 52 hours in Liquid Killing assay, colonization at time points later than 40 hours was not measured.

Other potential explanations for the reduced colonization in liquid included a difference in bacterial concentration (i.e. that a bacterial lawn functionally increases the concentration of the bacteria) or the richness of the media composition (i.e. Liquid Killing media is essentially Slow Killing media that has been diluted with non-nutritive volume). The Liquid Killing assay is inoculated with an initial bacterial inoculum of OD_600_ 0.03 to prevent excess oxygen consumption by the bacteria. Since this was not a concern on solid medium, worms were reared on a bacterial lawn with a considerably larger number of bacteria, according to established protocols [34–36]. To more rigorously test these possibilities, the initial *P. aeruginosa* concentration in liquid was tripled and colonization was assessed again. No significant change in colonization was detected under these conditions (**S1 Fig**). To determine whether dilution of Slow Killing media with S Basal drove the observed difference in colonization, we tested undiluted Slow Killing medium and undiluted BHI, which is even richer in nutrients. Neither media alteration had any apparent effect on host colonization (**S1 Fig**).

To gain a broader perspective on this phenomenon, we examined whether the lack of colonization in liquid is specific to *P. aeruginosa* or may reflect a more global outcome due to changes in the physiology of the host and/or the pathogen due to the liquid environment. *C. elegans* were exposed to *E. faecalis* OG1RF:GFP, either on agar or in liquid pathogenesis media for 24 hours, and then colonization was assessed via fluorescence. Although *E. faecalis* is generally more efficient at colonizing the intestine of *C. elegans* than *P. aeruginosa*, a strong reduction of colonization was also observed when the liquid assay was compared to the solid assay (Figure 1d–f). The same trend was seen for *C. albicans* (Figure 1g and h), a fungal pathogen that also colonizes the intestinal lumen of the worm [17]. Taken together, the decrease in colonization in liquid was independent of the pathogen, or even type of pathogen (i.e. Gram-negative or -positive bacteria or fungus), and did not appear to strongly depend on initial bacterial density or media type.

**Figure 2.**
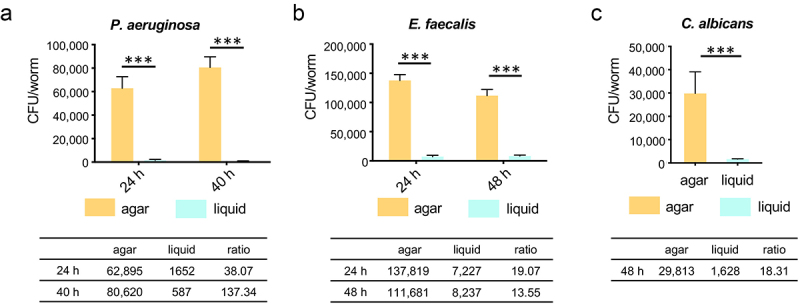
Worms in liquid exhibit reduced CFU for each pathogen. (a-c) Young adult worms were fed *P.* *aeruginosa*, *E.* *faecalis*, *E.* *faecium*, or *C.* *albicans* on agar or in liquid media, respectively. Worms were harvested at time points specified in figure panels. The average CFU per single worm was calculated. Each of the three biological replicates contained three technical replicates. Each of the technical replicates included 15 worms. Error bars represent SEM. *p* values were determined from Student’s *t*-test. ***p < 0.001.

The assays used above utilized the expression of fluorescent proteins by the pathogens as an indicator of colonization. While convenient, this approach can be hindered by the presence of intestinal autofluorescence in sick or senescent worms that can confound results [37–39]. It is also possible for fluorescence to remain in the host intestine from digested microbes, even in the absence of a viable pathogen. To more accurately quantify pathogen colonization under different infection conditions, we measured colony-forming units (CFUs) by homogenizing *C. elegans* to release internalized microbes [40]. In each case, colonization in the agar-based model was at least an order of magnitude higher than liquid (Figure 2a–c). Consistent with results from the fluorescent reporter, colonization by *P. aeruginosa* substantially increased during agar infection (from 24 to 48 hours), but not in liquid (Figure 2a). These results indicated that being in a liquid environment, rather than bacterial density or media composition, impaired colonization of *C. elegans*.

#### The liquid environment contributes to restricted pathogenic colonization of the host

**Figure 3.**
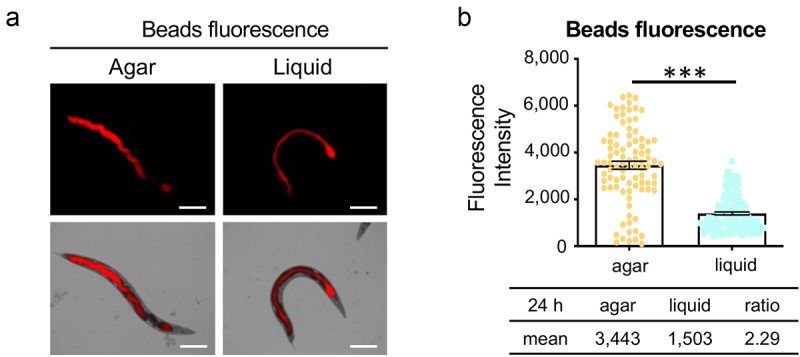
A liquid environment inhibits worm feeding. (a) Young adult worms were fed with fluorescent beads. Worms from agar and liquid media were imaged after 24 hours of incubation. Images show fluorescent beads in the intestinal lumen. Scale bar 200 μm. (b) Quantification of the fluorescence intensity in (a). Three biological replicates were performed with 50 worms for each replicate. Error bars represent SEM. *p* values were determined from Student’s *t*-test. ***p < 0.001.

We hypothesized that a liquid environment may decrease *C. elegans*’ ability to feed, indirectly limiting the potential for intestinal colonization [41]. To test this hypothesis, we assessed food uptake by measuring the accumulation of bacteria-sized (~1 µm) fluorescent beads in the intestinal lumen of *C. elegans*. In both agar- and liquid-based conditions, worms accumulated substantial fluorescent material in their intestinal tract (Figure 3a). Although worms fed on agar accumulated more fluorescent beads than those in liquid, the difference (~2.3-fold) was considerably lower than what was observed for bacterial colonization (~10-fold) Figure 1 and Figure 3. This indicated that although the liquid environment affects bacterial ingestion, it is not the main factor.

#### *P.* *aeruginosa* adhesins may have a weak impact on colonization in liquid

Adhesion to the surface of host tissue is a crucial step in colonization. Previous work demonstrated that mutations in adhesin genes led to colonization deficiency in *C*. *albicans* [42]. In *P. aeruginosa*, several cell membrane-anchored adhesins facilitate colonization [6], including cell appendages (e.g. type IV pilus) [43], exopolysaccharides (e.g. Pel, Psl, alginate) [44], and glycan-binding proteins (lectins) [45]. We assessed the expression of adhesins in *P. aeruginosa* cells growing on agar or in liquid media via qRT-PCR. Among the genes tested, only the expression of *pelA* and *pelB* was significantly decreased in the liquid setting (**S2 Fig**). PelA and PelB are involved in biosynthesis of the cationic polysaccharide Pel, a key component of extracellular matrix that facilitates bacterial aggregation [46]. We hypothesized that the downregulation of these genes in *P. aeruginosa* may contribute to the decrease in colonization. However, transposon insertions into *pelA* or *pelB* alone did not decrease colonization when worms were exposed to PA14*pelA* or PA14*pelB* (**S2 Fig**). In an orthogonal approach, instead of disrupting cell-cell adhesion, we induced the aggregation of planktonic cells by adding Pseudomonas quinolone signal (PQS), a small quorum-sensing molecule produced by *P. aeruginosa* [47]. While PQS supplementation increased planktonic aggregation (**S2 Fig**), it did not increase PA14:dsRed fluorescence within the worm, even after 40 hours of exposure (**S2 Fig**). Taken together, bacterial adhesion may play a role in colonization, but this appears to be minor and does not seem to be affected by the disruptions of individual adhesins.

#### C. elegans innate immune pathways only modestly affect colonization in liquid

**Figure 4.**
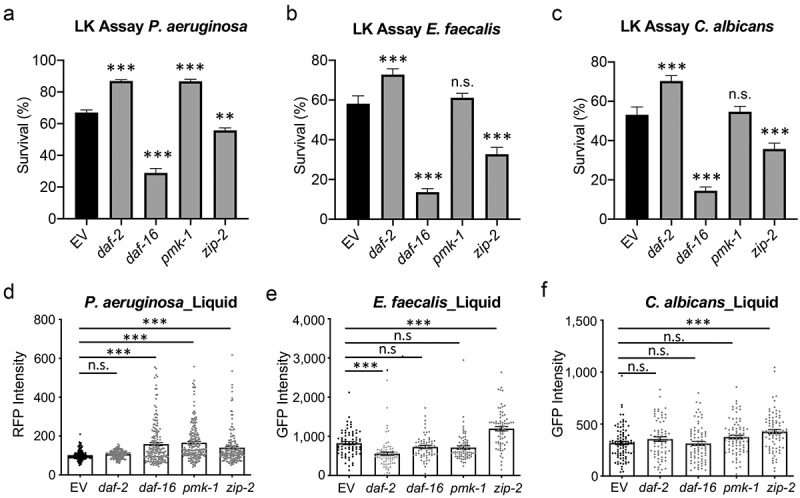
Disruption of host defense pathways had minimal effects on colonization. Young adult *glp-4(bn2)* worms reared on RNAi were transferred into PA14:dsRed, OG1RF:GFP, or fRS26:GFP in liquid media. (a-c) Survival was measured using a cell-impermeant nucleic acid stain after 55 hours (*P.* *aeruginosa*, A) or 5 days (*E.* *faecalis* (b) or *C.* *albicans* (c)). (d-f) the fluorescence of worms was measured after 24 hours to quantify colonization. Three biological replicates, each with 400 worms (a-c) or with 50 worms (d-f) were performed for each replicate. Error bars represent SEM. *p* values were determined from one-way ANOVA, followed by Dunnett’s test. n.s. *p* > 0.05; ***p < 0.001.

Maintenance in liquid culture has been associated with the activation of several innate immune genes in *C. elegans* [48]. To investigate whether the activation of immune pathways restricted colonization in liquid, and to assess the relationship between colonization and host survival, we measured *C. elegans* death and bacterial CFUs in worms where RNAi was used to knock down innate immune pathways. As we previously reported for *P. aeruginosa* [49], insulin signaling and ZIP-2 pathways were required for host survival during exposure to multiple pathogens in liquid, while PMK-1 was either dispensable for the host defense or was detrimental (Figure 4a–c). Statistically-significant increases in bacterial colonization were observed in *zip-2(RNAi)* animals for all three pathogens and for *daf-16 (RNAi)* in the case of *P. aeruginosa* (Figure 4d–f). However, the fold-change of this increase was rather modest and did not reflect the dramatic decrease in colonization seen upon the switch from agar to liquid infection models.

**Figure 5.**
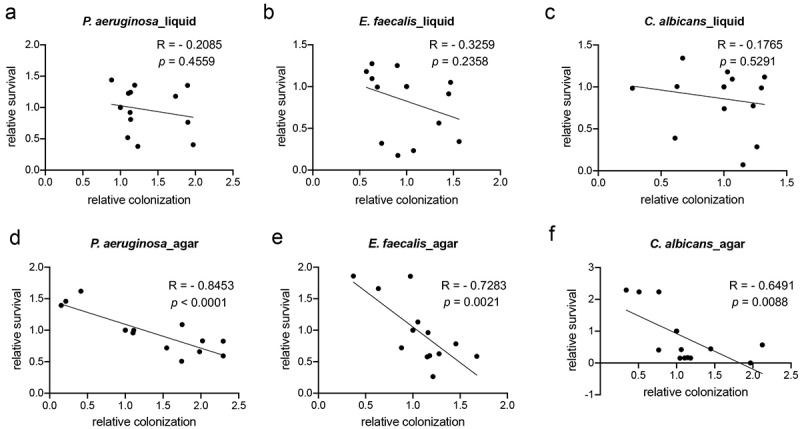
Pathogen virulence is colonization-dependent on agar plate but not in liquid. Young adult *glp-4(bn2)* worms were reared on empty vector, *daf-2*, *daf-16*, *pmk-1*, or *zip-2* RNAi and then exposed to *P.* *aeruginosa* PA14:dsRed, *E.* *faecalis* OGRF1:GFP, or *C.* *albicans* fRS26:GFP in liquid- (a) or agar-based (b) contexts. Host survival and pathogenic colonization for each combination of RNAi (*daf-2*, *daf-16*, *pmk-1*, or *zip-2*) and pathogen (*P.* *aeruginosa*, *E.* *faecalis*, and *C.* *albicans*) were normalized to their empty vector group control. Data shown include all three biological replicates for each group. The relative survival and colonization were plotted. R and *p* values were determined from Pearson correlation.

To address the possibility of genetic redundancy, we took advantage of the observation that knocking down either *daf-16* or *zip-2* decreased *C. elegans* survival and slightly increased bacterial colonization. We assessed host survival and bacterial colonization in a *daf-16(RNAi)*; *zip-2(tm4248)* double mutant background. Since *E. faecalis* more effectively colonizes *C. elegans* than *P. aeruginosa*, we postulated that it may more effectively take advantage of the weakened immune system. Survival of the double mutant was less than half of single mutants, but colonization in the double mutant was only increased by ~14% (**S3 Fig**). In parallel, we performed analogous experiments using agar-based infection models (**S4 Fig**). As expected, we saw no correlation between colonization and killing in liquid media, but a statistically-significant negative correlation between colonization and worm survival for all three pathogens tested on agar (Figure 5). Taken together, our data suggest that colonization is not the main driver of host death in liquid-based pathogenesis models.

### Forced colonization can enhance the virulence of *E.* *faecalis* in liquid

**Figure 6.**
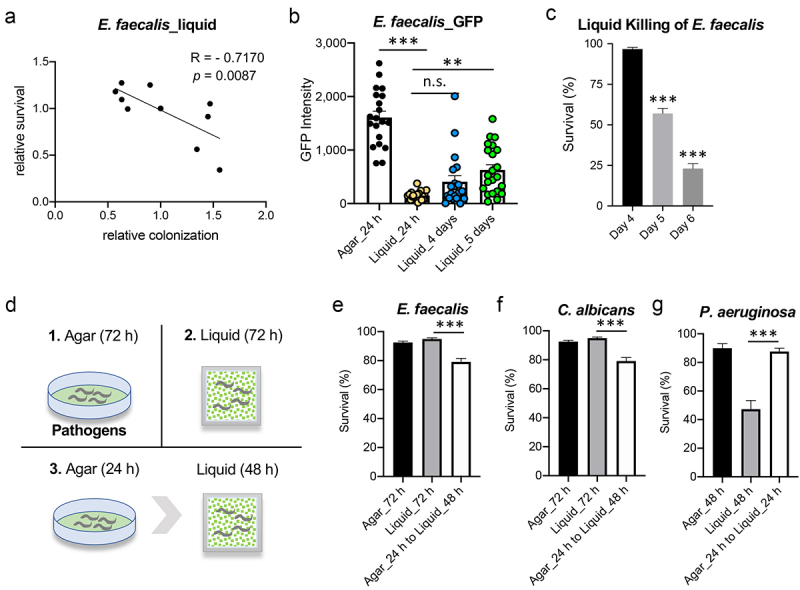
Intestinal colonization contributes to *E.* *faecalis*- and *C.* *albicans*-mediated killing in liquid. (a) Data from Figure 5b were replotted without data from *daf-16(RNAi)* worms. (b) Intestinal fluorescence of worms exposed to *E.* *faecalis* OG1RF:GFP on agar or in liquid. (c) Survival of young adult *glp-4* worms infected with *E.* *faecalis* OG1RF in liquid after 4–6 days. (d) a schematic illustrating infection conditions for panels (e-g). (e-g) Survival of young adult glp-4 worms infected with E. faecalis OG1RF (e), *C.* *albicans* (f), or *P.* *aeruginosa* (g) for 24 hours on agar and then transferred to liquid containing the same pathogen for 48 hours (e, f) or 24 hours (g). Also shown are young adult *glp-4(bn2)* worms exposed to *E.* *faecalis* (e), *C.* *albicans* (f), or *P.* *aeruginosa* (g) for 72 hours (e, f) or 48 hours (g) on agar or in liquid without transfer. (b-g) Data indicate results from three biological replicates, each with 30 (b) or 400 (c-g) worms. Error bars represent SEM. *p* values were determined from one-way ANOVA, followed by Dunnett’s test. n.s. *p* > 0.05; * *p* < 0.05; ** *p* < 0.01; *** *p *< 0.001.

In liquid, *P. aeruginosa* disrupts iron homeostasis and kills the host in part through the activity of a secreted siderophore called pyoverdine [15]. *C. albicans* undergoes a morphological change to form hyphae, which results in tissue destruction and worm death [17]. The mechanism for *E. faecalis* pathogenesis in liquid remains unclear. However, we noticed that removing the RNAi data for *daf-16(RNAi)* revealed a significant negative correlation between colonization and host survival in worms exposed to *E. faecalis* in liquid (Figure 6a). This phenomenon was not observed for *P. aeruginosa* or *C. albicans* (**S5 Fig**), suggesting that colonization may contribute to the pathogenesis of *E. faecalis* in some contexts. To follow up on this observation, we measured colonization and host survival over time. For the first 96 hours, no significant accumulation of fluorescence was seen in *C. elegans* in the liquid assay with *E. faecalis* OG1RF:GFP. By day 5, however, an increase in GFP accumulation was recorded (Figure 6b). Increased host mortality was seen on days 5 and 6 (Figure 6c). To determine whether increased colonization impacted pathogenesis, we first reared *C. elegans* on a lawn of *E. faecalis* on agar plates for 24 hours to promote intestinal colonization. Afterward, worms were transferred to liquid media containing *E. faecalis* for 2 days (Figure 6d). Compared to the liquid-only condition, worms pre-colonized on the agar exhibited increased death (Figure 6e), suggesting that colonization contributes to *E. faecalis* pathogenesis. Pre-colonization also increased lethality for *C. albicans* but the same effect was not seen for *P. aeruginosa* (Figure 6f–g). This is consistent with reported findings that *E. faecalis* and *C. albicans* cause intestinal destruction, while *P. aeruginosa* kills worms by secreting virulence factors into the medium [15,17].

To test whether *E. faecalis* virulence also depended upon a secreted factor, we collected spent, bacteria-free growth media from the liquid-based assay for *E. faecalis* after 3 days and tested whether it increased the rate of worm killing in the liquid assay. The literature indicated that *E. faecalis* releases potential virulence factors like gelatinase, cytolysin, polysaccharides, and other surface proteins during biofilm formation [50]. Therefore, we also collected supernatant after 6 days of incubation, in case the robust biofilms that developed after 4 days stimulated the secretion of a pathogenic factor. Adding spent media to the assay had no apparent effect on host killing in either case (**S6 Fig**), suggesting that secreted factors are not involved in this killing assay.

### Host-pathogen interactions contribute to bacterial clearance in liquid

**Figure 7.**
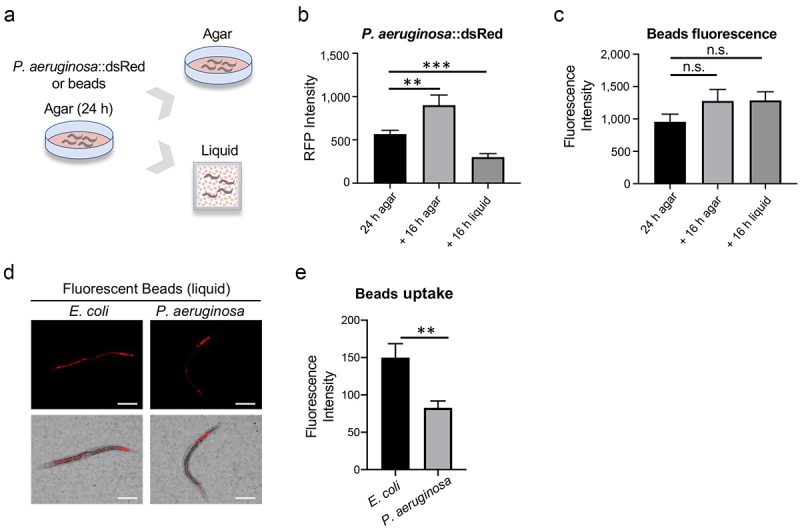
Transfer to liquid can stimulate clearance of *P.* *aeruginosa* from the host. (a) a schematic diagram illustrating the experimental design for panels (b) and (c). (b, c) Quantification of intestinal fluorescence from worms fed *P.* *aeruginosa* PA14:dsRed (b) or beads (c) on agar plates. After 24 hours worms were split and transferred to liquid media or new agar plates containing *P.* *aeruginosa* PA14:dsRed (b) or beads (c) for an additional 16 hours. (d) Fluorescent and transmitted light images of worms in Liquid Killing assays containing fluorescent beads and either *E.* *coli* OP50 or *P.* *aeruginosa* PA14 after 24 h. (e) Quantification of fluorescence from worms treated as in (d). At least three biological replicates, comprised of 50 worms each, were used. Error bars represent SEM. *p* values were determined from one-way ANOVA, followed by Dunnett’s test or Student’s *t*-test. n.s. *p* > 0.05; **p < 0.01; ***p < 0.001.

One possible explanation for the difference between bacterial colonization and accumulation of fluorescent beads in the intestine is that the microbial species are likely to interact with the host, while fluorescent beads would not. To test this, we first incubated worms on a bacterial lawn on agar plates with dsRed-labeled *P. aeruginosa* PA14 for 24 hours before transferring them to agar or liquid medium in the presence of PA14:dsRed for an additional 16 hours (Figure 7a). A control experiment was performed in parallel where fluorescent beads were substituted for bacteria. This allowed the intestine to be pre-loaded with bacteria or beads. As expected, the intestinal fluorescence of worms transferred to fresh bacteria on agar continued to increase. In contrast, worms pre-colonized with PA14:dsRed bacteria lost intestinal fluorescence after being transferred to liquid (Figure 7b). Worms fed with fluorescent beads remained essentially unchanged, regardless of which medium they were transferred to (Figure 7c). These findings indicate that some combination of the live pathogen and the liquid medium triggers host responses that clear bacterial colonization.

*P. aeruginosa* utilizes several secretion systems to release host-damaging factors [32]. We posited that one or more of these factors may adversely affect host physiology, such as by impeding pharyngeal pumping. To test this hypothesis, we exposed *C. elegans* to a mixture of *P. aeruginosa* and fluorescent beads or a mixture of *E. coli* and fluorescent beads in liquid. Notably, we observed significantly decreased bead uptake in worms exposed to *P. aeruginosa* (Figure 7d–e), confirming that *P. aeruginosa* exposure decreased pharyngeal pumping.

#### *C. elegans* become dormant after exposure to pathogens in liquid

**Figure 8.**
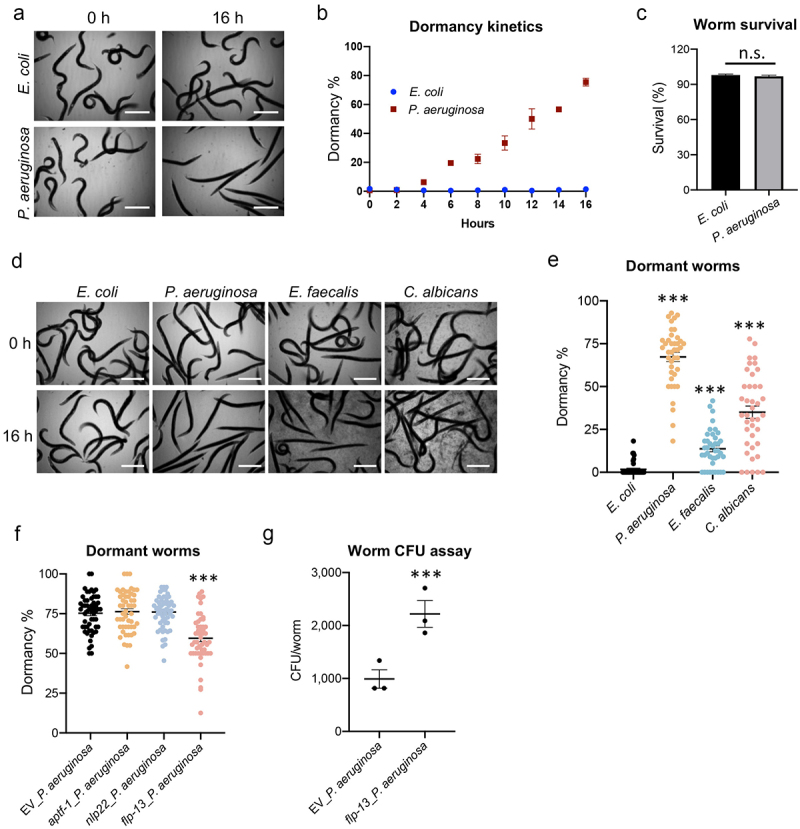
Pathogen-induced dormancy limits infection. (a-c) Young adult *glp-4(bn2)* worms were subjected to Liquid Killing assays with either *E.* *coli* OP50 or *P.* *aeruginosa* PA14 and transmitted light images were collected every 2 hours for 16 hours. (a) Representative micrographs of worms in Liquid Killing assays after 0 and 16 hours of exposure to *E.* *coli* OP50 (top) or *P.* *aeruginosa* PA14 (bottom). (b) Quantification of worms that have entered a dormant state, as determined by visual analysis. (c) Quantification of survival, as measured by a cell-impermeant nucleic acid stain, after 16 hours of Liquid Killing. (d-f) Young adult *glp-4(bn2)* worms were subjected to liquid-based killing assays for *E.* *coli*, *P.* *aeruginosa*, *E.* *faecalis*, or *C.* *albicans* and worms were imaged at 0 and 16 hours. (e) Quantification of worms exhibiting a dormant phenotype after exposure to each pathogen for 16 hours. (f) Quantification of dormancy in *glp-4(bn2)* worms reared on RNAi (empty vector (EV), *aptf-1*, *nlp-22*, or *flp-13*) after 16 hours of exposure to *P.* *aeruginosa* in Liquid Killing. (g) Bacterial titer from *glp-4(bn2)* worms reared on empty vector (EV) or *flp-13(RNAi)*. Data shown are mean values for three biological replicates, each comprised of 20 technical replicates of 20 worms (b, c, e, f), and mean values for three technical replicates of 15 worms (g). Error bars represent SEM. *p* values were determined from one-way ANOVA, followed by Dunnett’s test or Student’s *t*-test. n.s. *p* > 0.05; ***p < 0.001.

We also noticed after 16 hours that a substantial fraction of worms exposed to *P. aeruginosa* in liquid became dormant, assuming a rigid, rod-like posture, while worms incubated with *E. coli* remained sinuous and active (Figure 8a). Time course experiments indicated that this dormancy started as early as 4–6 hours into the incubation (Figure 8b). These observations were consistent with the lethargic pumping observed earlier. We measured the viability of these worms using a cell-impermeant nucleic acid stain and confirmed that, despite their dormancy, > 90% of the worms exposed to *P. aeruginosa* were still alive at 16 hours, a viability similar to *E. coli* controls (Figure 8c). Worms incubated with *E. faecalis* or *C. albicans* also displayed dormancy, albeit to a lesser extent (Figure 8d–e). This quiescence, which has been referred to as “sleep” by some groups, had been described as a host defense mechanism to extend survival under stress [51]. Rescue of the rigid phenotype was observed in worms when the sleep-related gene *flp-13* was disrupted by RNAi (Figure 8f), indicating that this dormancy may be an adaptive response by the host to limit pathogen ingestion and colonization. Consistent with this interpretation, *flp-13(RNAi)* worms had significantly increased intestinal colonization with *P. aeruginosa* (Figure 8g).

## Discussion

To date, *C. elegans* is the only commonly-used metazoan animal that can be used in pathogenesis models both on a solid and a liquid media [14]. Liquid-based *C. elegans* pathogenesis models have been reported for a range of bacteria, including *P. aeruginosa* [52], *Staphylococcus aureus* [53], and *Acinetobacter baumanii* [54]. The adaptability and cost-effectiveness of these models make them an obvious choice for high-content, high-throughput screening efforts [16], and their use is likely to increase as the search for new methods to treat bacterial infections continues. However, these assays are likely to be more complicated than is immediately apparent. For example, the first step of establishing infection is typically colonization; this is true in *C. elegans* as with all organisms. On agar plates, this is often as simple as replacing the normal bacterial foodstuff—*E. coli* OP50—with the pathogen of interest.

In the earliest attempts at using *C. elegans* for screening for anti-infectives, a hybrid mode was used that involved pre-infecting worms on agar prior to transferring the worms to liquid [17,18,55,56]. This step was long regarded as a necessary pre-requisite for liquid-based drug screening. Although this step is can be helpful, it is likely to change the nature of the host-pathogen interaction, and should be carefully considered during experimental design and interpretation.

Colonization in liquid was strongly attenuated, independently of which pathogen we tested. Indirect assessment of food uptake using fluorescent beads demonstrated that worms consumed about half as many beads in a liquid environment than on solid. There are several potential explanations for this, including locomotory fatigue and energy exhaustion in a fluid environment [57] and observations that feeding is reduced when *C. elegans* is stressed or in liquid [58,59]. This latter explanation is consistent with our observations that DAF-16, a common stress marker in *C. elegans*, was upregulated in all of the liquid assays tested.

Due to the innate immune functions of the insulin signaling pathway [60–62], its activation by the liquid environment triggers a constitutive pro-survival response that enhances host defense against all three pathogens we tested, as can be seen by the deleterious effects of *daf-16(RNAi)*. Importantly, this is different from observations of agar-based assays, where *daf-16* knockdown or mutation did not increase host mortality, despite the strong survival benefit conferred by artificial activation of DAF-16 by *daf-2(RNAi)* [63].

In contrast, knockdown of *pmk-1*/p38 MAPK, another key innate immune pathway in *C. elegans*, did not impact host survival in liquid after infection with *E. faecalis* or *C. albicans*. Interestingly, *pmk-1(RNAi)* increased survival in the liquid *P. aeruginosa* assay, which was consistent with previous reports that p38/MAPK activity was deleterious for survival in Liquid Killing, possibly due to the high energy costs of producing the secreted effectors downstream of PMK-1 [49] while worms are undergoing a metabolic crisis from mitochondrial damage [49].

The liquid assays that we studied in this report kill the host in spite of poor colonization, and often do so in significantly shorter timespans than their solid counterparts [15,64]. The available evidence suggests that killing in liquid models frequently uses pathogenic determinants that are characteristic of acute infection. For example, we have shown that *P. aeruginosa* killing depends on production of pyoverdine. A previous report showed that *C. albicans* infection in the liquid setting results in a morphologic shift in the pathogen, promoting hyphal growth that pierces worms’ tissues [17]. Loss of hyphal growth was reported to attenuate killing in both *C. elegans* and in mammals [17,65]. Although there has been considerable study of the host response to *E. faecalis* and some virulence determinants in *C. elegans* have been identified [34,66–68], much of this work has been done on agar. At least one acute virulence factor, the protease cytolysin, is shared by *C. elegans* and mammalian models [34], but further study will be necessary to evaluate whether this factor is important in this liquid assay. Generally speaking, liquid-based infection models appear to effectively recapitulate many aspects of acute pathogenesis in mammals. This is in contrast to assays on solid media, which often involve more chronic aspects of virulence. This suggests that liquid assays may also serve as a valuable tool for decoding the acute-chronic switch since the pathogenic colonization is only established on the agar-based model, not in the liquid-based model.

We observed that worms entered dormancy early in liquid-based pathogenesis models, but not during incubation with *E. coli* in liquid. This was consistent with previous reports that *C. elegans* sometimes responds to stress by entering a quiescent state [27,69]. Notably, those worms retained their curved shape, which is different from the rigid, rod-like shape observed in our infection condition, suggesting a significant departure from other stress-driven quiescence pathways. In addition, this stress-induced quiescence is relatively short compared to the pathogen-driven dormancy we observed. One potential explanation is that the broad-spectrum toxins secreted by *P. aeruginosa* may contribute to this dormancy phenotype, but this remains to be evaluated. As noted, *C. elegans* enters a long-term dormant state when confronting an unfavorable environment (i.e. the dauer state), but this transition is only known to occur in larval worms entering an alternative L2d stage instead of the canonical L2 stage [26]. Whether certain features are shared by pathogen-induced and “dauer” dormancy needs to be investigated.

We demonstrated that this quiescence pathway was protective. Mutation of *flp-13* diminished quiescence and increased colonization, linking the two phenotypes. FLP-13 belongs to the FMRFamide-like peptide family of neuropeptides and is released by ALA neurons, which regulate sensory processing, locomotion, and feeding behavior based on different stimuli [28,70]. FLP-13 has been shown to be involved in the regulation of locomotion, specifically in the modulation of the rate of the body waves and is required for stress-induced recovery quiescence [71]. How the host recognizes the pathogen and delivers a signal to the ALA neuron to trigger FLP-13 release to induce quiescence is an open question for future study.

## Supplementary Material

Supplemental Material

## Data Availability

The authors confirm that the data supporting the findings of this study are available within the article and its supplementary materials.
