# Supplementary material for: Pathogen-induced dormancy in liquid limits gastrointestinal colonization of Caenorhabditis elegans
Source: Virulence. 2023 Apr 25;14(1):2204004. doi: 10.1080/21505594.2023.2204004 (PMC10132241; doi:10.1080/21505594.2023.2204004)
Supplement: Supplemental Material [file KVIR_A_2204004_SM4267.zip › Supplemental_Figure_Legends.docx]

**Supporting Figures**

**S1 Fig. Bacteria concentration and medium composition are not factors affecting colonization.** (**A-C**) Average worm CFU was determined with an initial OD_600_ of 0.03 and 0.09 (**A**), with 30% and 100% SK (**B**), and with 10% BHI (**C**) under the infection of *P. aeruginosa* in the liquid medium. Each of the three biological replicates included three technical replicates. Each of the technical replicates included 15 worms. Error bars represent SEM. *p* values were determined from Student’s t-test. n.s. *p*>0.05.

**S2 Fig. Pathogen plays a trivial role in the decrease of colonization in liquid.** (**A**) Gene expression levels of seven adhesin genes (*pilI, pilJ, pela, pelB, pa1L,* and *lecB*) normalized to the *P. aeruginosa* housekeeping gene *rpsL* for *P. aeruginosa* under both liquid and agar conditions was measured via qRT-PCR. (**B**) The average worm CFU of wildtype, *pelA*, and *pelB* mutant *P. aeruginosa* was measured after 24-hour infection on agar plates. Each of the three biological replicates contained three technical replicates. Each of the technical replicates included 15 worms. (**C**) The pictures of *P. aeruginosa* were captured under the treatment of PQS and DMSO (negative control) in SK medium after 24 hours of incubation (scale bar 500 μm). (**D-F**) Worm fluorescence (scale bar 500 μm) and quantification under treatment of PQS and DMSO (negative control) were captured and determined after 24 or 40 hours of infection. Three biological replicates were performed with 50 worms for each replicate. Error bars represent SEM. *p* values were determined from one-way ANOVA, followed by Dunnett’s test or Student’s t-test. n.s. *p* > 0.05; **p* < 0.05.

**S3 Fig. DAF-16 and ZIP-2 protect worms from pathogenic killing.** (A) Survival of RNAi-treated worms on the *zip-2(tm4248)* mutant background in the infection of *E. faecalis* OG1RF::GFP. Three biological replicates were performed with 400 worms for each replicate. (B) GFP intensity of RNAi-treated worms on the *zip-2(tm4248)* mutant background in the infection of *E. faecalis* OG1RF::GFP compared to worms from agar-based infection model. Three biological replicates were performed with 50 worms for each replicate. Error bars represent SEM. *p* values were determined from one-way ANOVA, followed by Dunnett’s test. n.s. *p>*0.05; **p*<0.05; ***p*<0.01; ****p*<0.001.

**S4 Fig. Host defense is active during infection on agar.** (A-C) Larva 1 stage *glp-4* worms were reared at RNAi bacterial lawn (EV (empty vector), *daf-2, daf-16, pmk-1, zip-2*). RNAi-treated worms were transferred onto the pathogen lawn. Dead worms were picked from plates and were recorded every day. Worm survival was displayed under infection of *P. aeruginosa, E. faecalis,* and *C. albicans.* Each of the three biological replicates contained three technical replicates. Each of the technical replicates had 150 worms. *p* values were determined from the log-rank test. Error bars represent SEM. n.s. *p>*0.05; * *p*<0.05; ** *p*<0.01; *** *p*<0.001.

**S5 Fig. The virulence of *P. aeruginosa* and *C. albicans* is not colonization-dependent in the liquid.** Host survival and pathogenic colonization of each RNAi group (empty vector, *daf-2, pmk-1, or zip-2*) were normalized to the empty vector group, respectively. Data were from all three biological replicates for each group. The relative survival and colonization were plotted. R and *p* values were determined from the Pearson correlation.

**S6 Fig. *E. faecalis* supernatant is not toxic to worms.** (**A, B**) The supernatant obtained from day 3 (**D**) and day 6 (**E**) of the liquid-based *E. faecalis* infection assay was tested on worm killing effect. The host survival was determined after 5 days of infection. Three biological replicates were performed with 400 worms for each group in each replicate. Error bars represent SEM. *p* values were determined from one-way ANOVA, followed by Dunnett’s test or Student’s t-test. n.s. *p>*0.05.
