# Supplementary figures and images for: Pathogen-induced dormancy in liquid limits gastrointestinal colonization of Caenorhabditis elegans
Source: Virulence. 2023 Apr 25;14(1):2204004. doi: 10.1080/21505594.2023.2204004 (PMC10132241; doi:10.1080/21505594.2023.2204004)

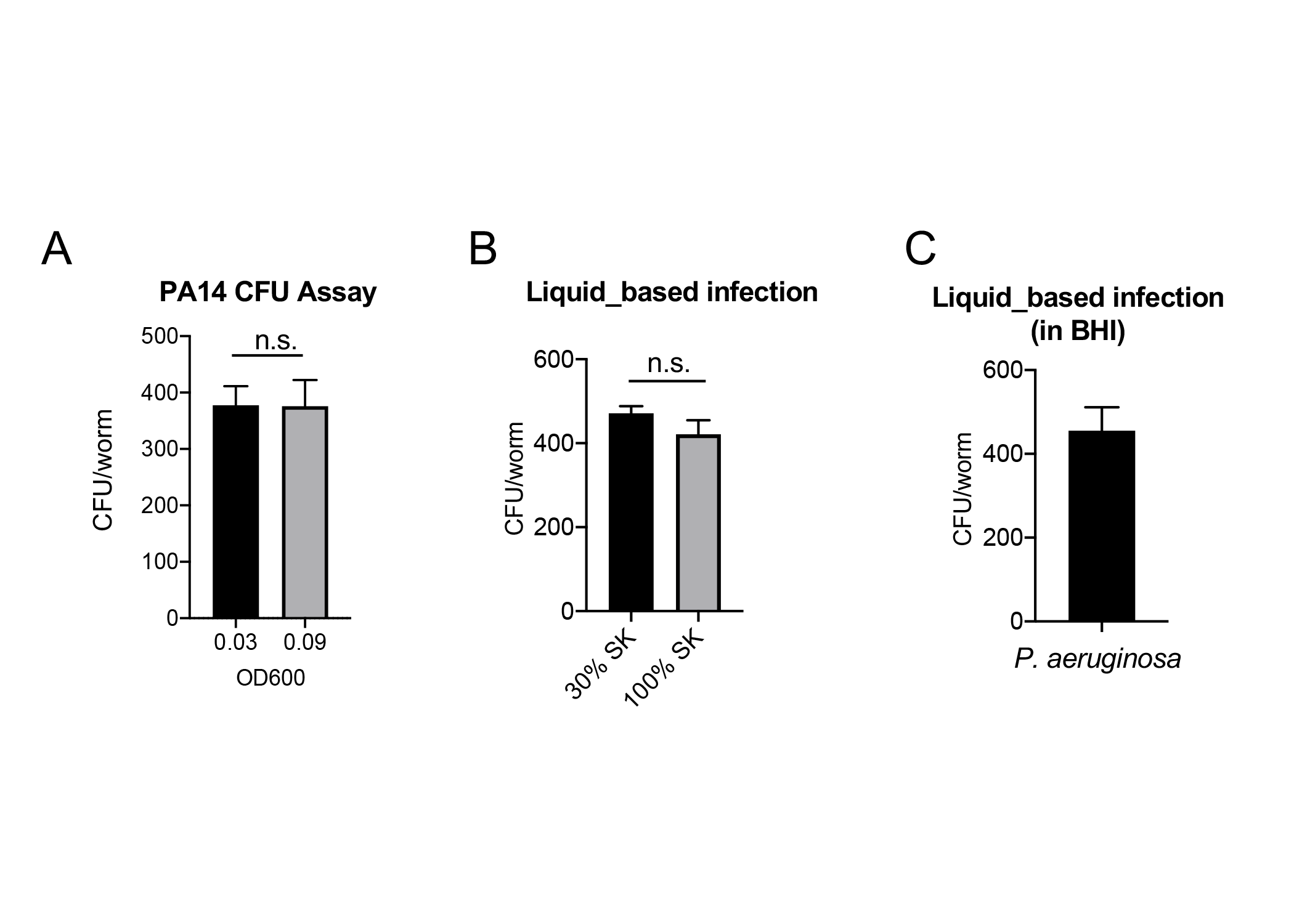

Supplement: Supplemental Material [file KVIR_A_2204004_SM4267.zip › Figure_S1 (1).tif]

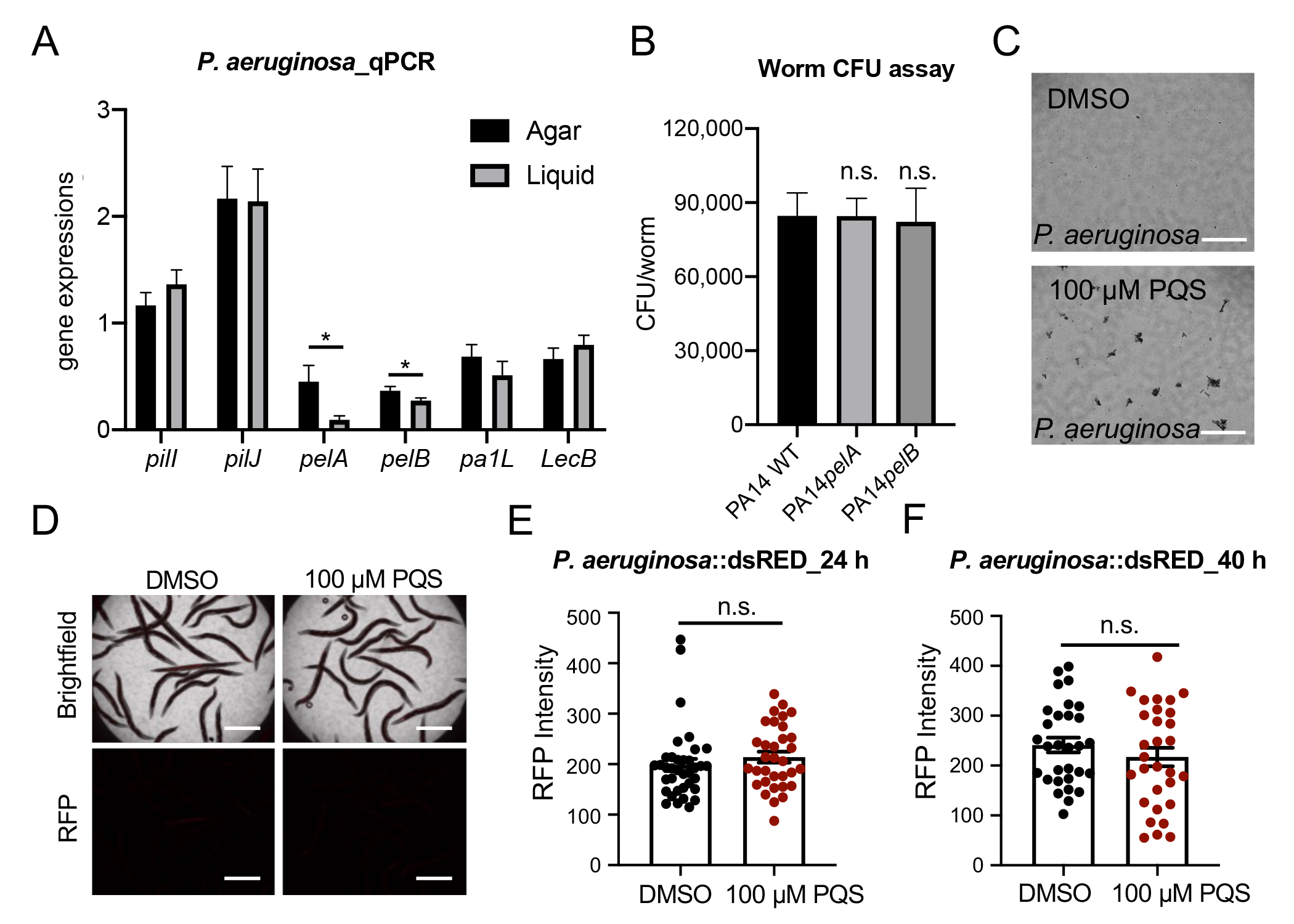

Supplement: Supplemental Material [file KVIR_A_2204004_SM4267.zip › Figure_S2 (1).tif]

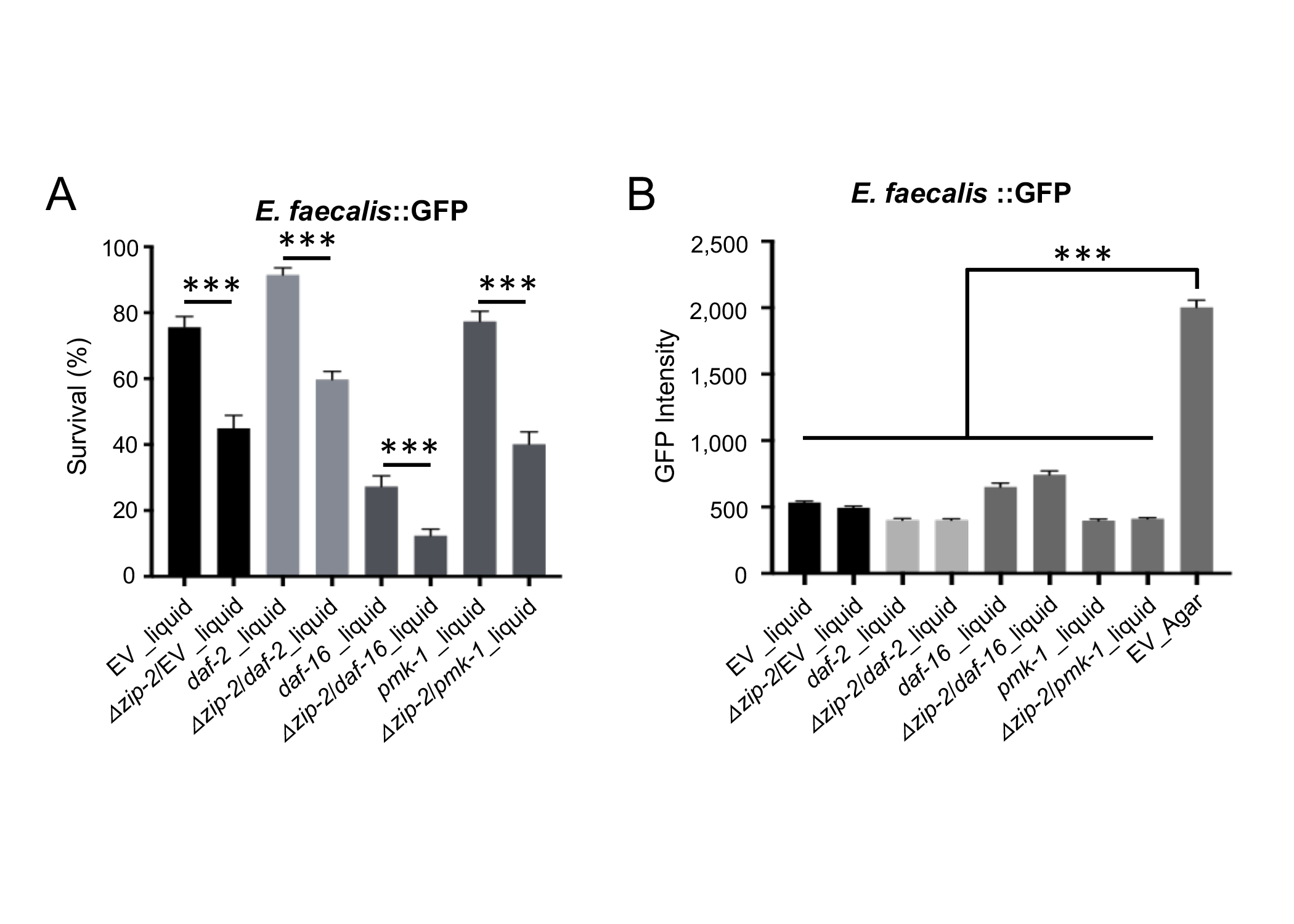

Supplement: Supplemental Material [file KVIR_A_2204004_SM4267.zip › Figure_S3 (1).tif]

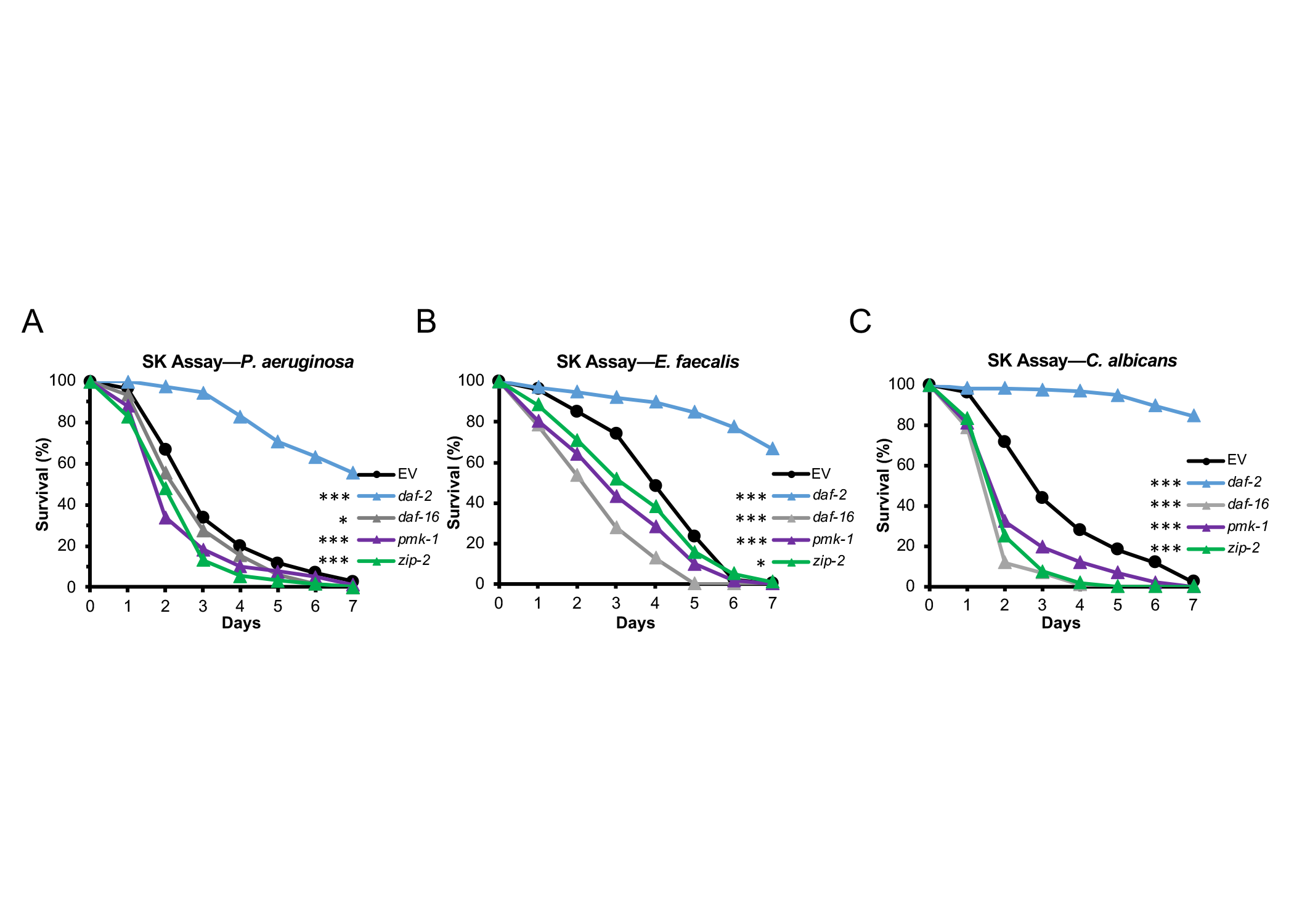

Supplement: Supplemental Material [file KVIR_A_2204004_SM4267.zip › Figure_S4 (1).tif]

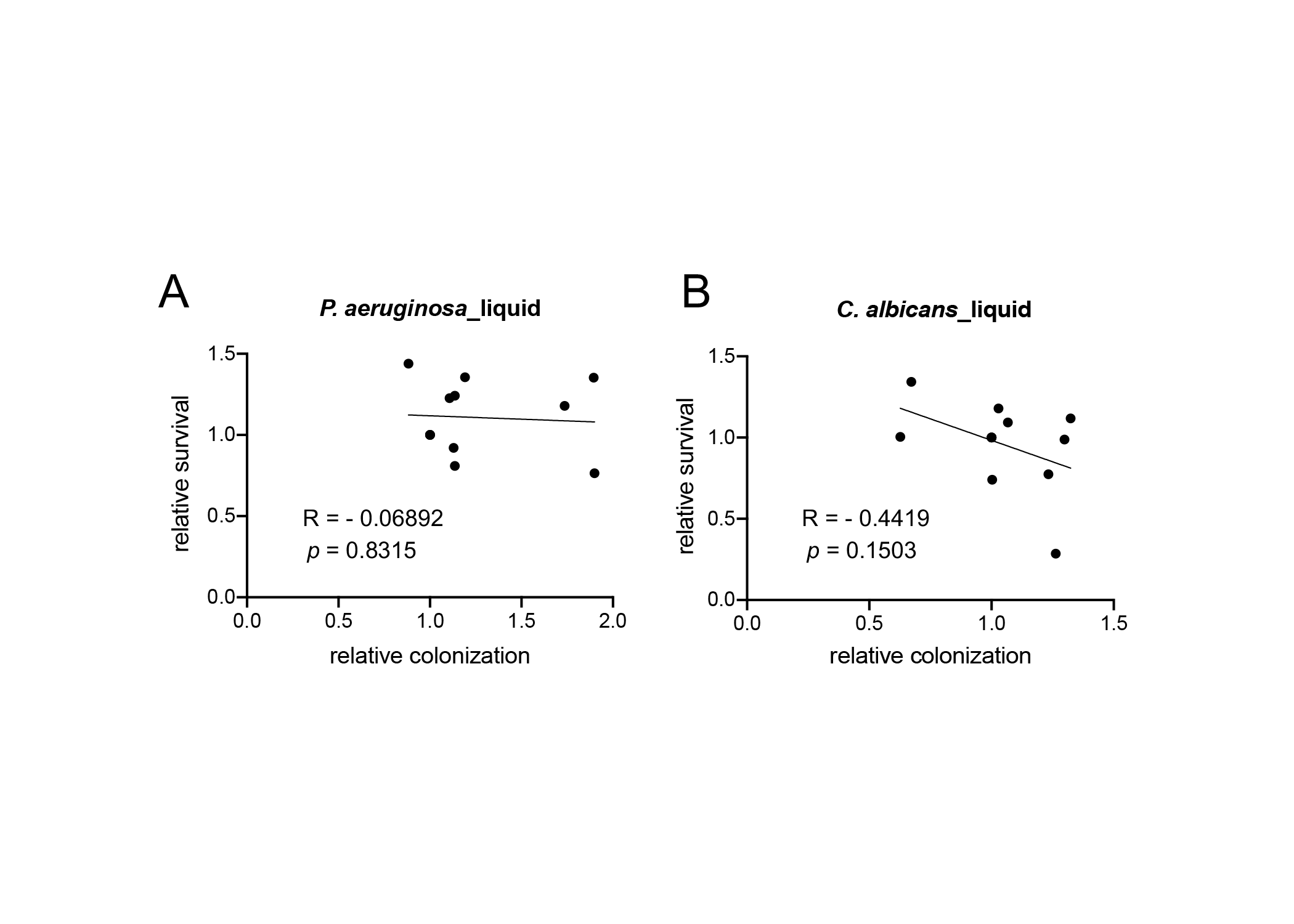

Supplement: Supplemental Material [file KVIR_A_2204004_SM4267.zip › Figure_S5 (1).tif]

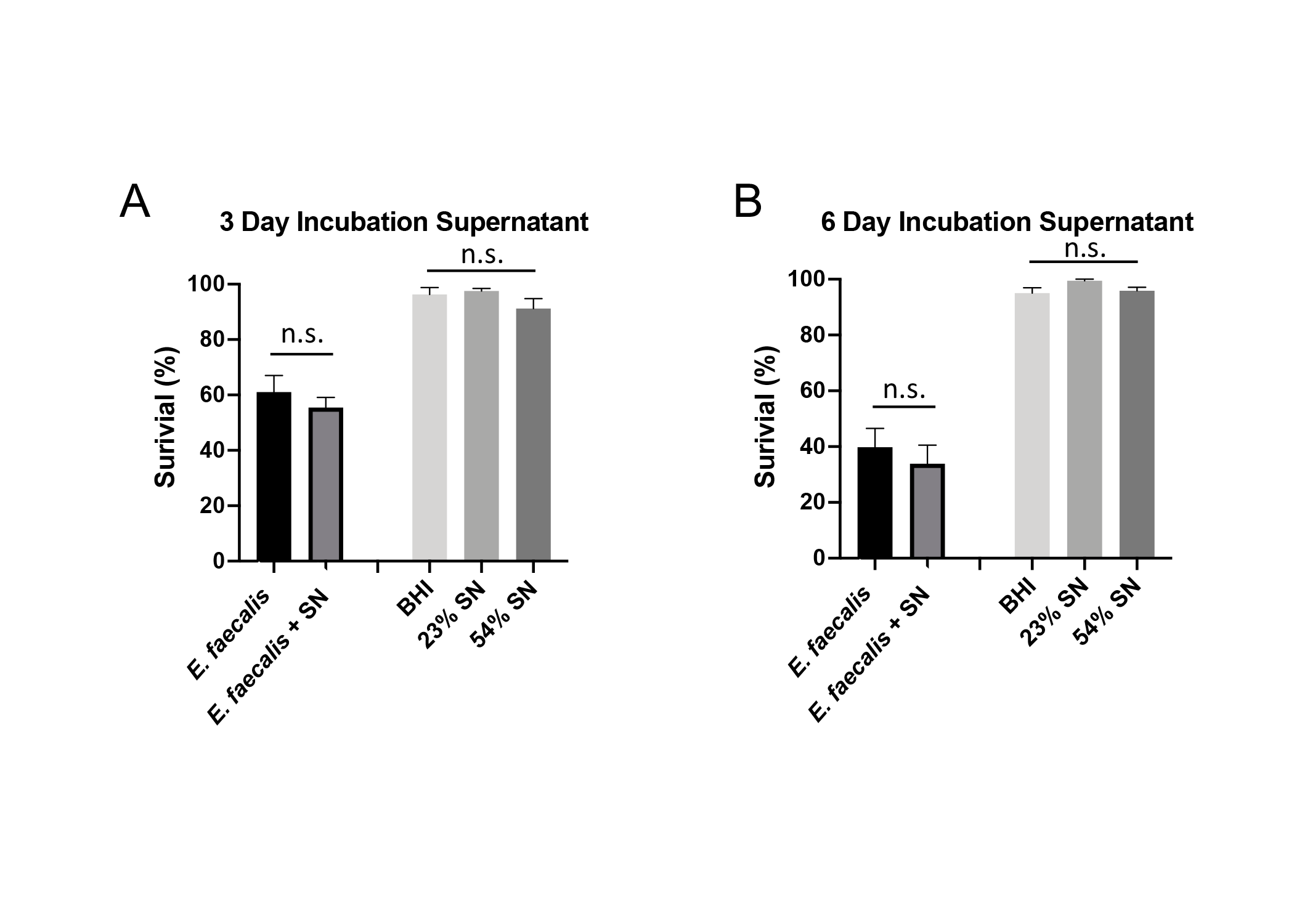

Supplement: Supplemental Material [file KVIR_A_2204004_SM4267.zip › Figure_S6.tif]
